# ChatGPT in Radiology: The Advantages and Limitations of Artificial Intelligence for Medical Imaging Diagnosis

**DOI:** 10.7759/cureus.41435

**Published:** 2023-07-06

**Authors:** Samriddhi Srivastav, Rashi Chandrakar, Shalvi Gupta, Vaishnavi Babhulkar, Sristy Agrawal, Arpita Jaiswal, Roshan Prasad, Mayur B Wanjari

**Affiliations:** 1 Medicine, Jawaharlal Nehru Medical College, Datta Meghe Institute of Higher Education & Research, Wardha, IND; 2 Surgery, Jawaharlal Nehru Medical College, Datta Meghe Institute of Higher Education & Research, Wardha, IND; 3 Obstetrics and Gynaecology, Jawaharlal Nehru Medical College, Datta Meghe Institute of Higher Education & Research, Wardha, IND; 4 Medicine and Surgery, Jawaharlal Nehru Medical College, Datta Meghe Institute of Higher Education & Research, Wardha, IND; 5 Research and Development, Jawaharlal Nehru Medical College, Datta Meghe Institute of Higher Education & Research, Wardha, IND

**Keywords:** limitations, advantages, medical imaging diagnosis, artificial intelligence, radiology, chatgpt

## Abstract

This review article provides an overview of using artificial intelligence (AI) in radiology. It discusses the advantages and limitations of ChatGPT, a large language model, for medical imaging diagnosis. ChatGPT has shown great promise in improving the accuracy and efficiency of radiological diagnoses by reducing interpretation variability and errors and improving workflow efficiency. However, there are also limitations, including the need for high-quality training data, ethical considerations, and further research and development to improve its performance and usability. Despite these challenges, ChatGPT has the potential to significantly impact radiology and medical imaging diagnosis. The review article highlights the need for continued research and development, coupled with ethical and regulatory considerations, to ensure that ChatGPT is used to its full potential in improving radiological diagnoses and patient care.

## Introduction and background

Radiology plays a crucial role in diagnosing and treating many medical conditions, providing physicians with images of the body's internal structures to aid in diagnosis and treatment planning. However, interpreting medical images is a complex and time-consuming process, often requiring specialized expertise and extensive training. In recent years, artificial intelligence (AI) has emerged as a powerful tool in radiology, offering new possibilities for improving accuracy, efficiency, and patient outcomes. One of the most promising AI technologies for medical imaging diagnosis is ChatGPT, a large language model trained by OpenAI that uses natural language processing (NLP) to analyze and interpret medical images [1,2].

ChatGPT has the potential to revolutionize the field of radiology by providing a more efficient and accurate way to diagnose medical images. Its ability to analyze and interpret images in real time could significantly reduce the time and resources required for diagnosis, improving patient outcomes and reducing healthcare costs [3,4].

The aim of this eview is to provide a comprehensive overview of the current state of the art in AI for medical imaging diagnosis, with a specific focus on the role of ChatGPT technology in radiology. The review will explore the advantages and limitations of AI for medical imaging, including the potential benefits of using ChatGPT for image interpretation, classification, and diagnosis. The review will also discuss the challenges and limitations of AI for medical imaging, including issues related to data quality, bias, and the need for human oversight. Ultimately, the goal of the review is to provide insights into the potential of ChatGPT technology to improve medical imaging diagnosis and inform future research in this area.

## Review

Methodology

**Figure 1:**
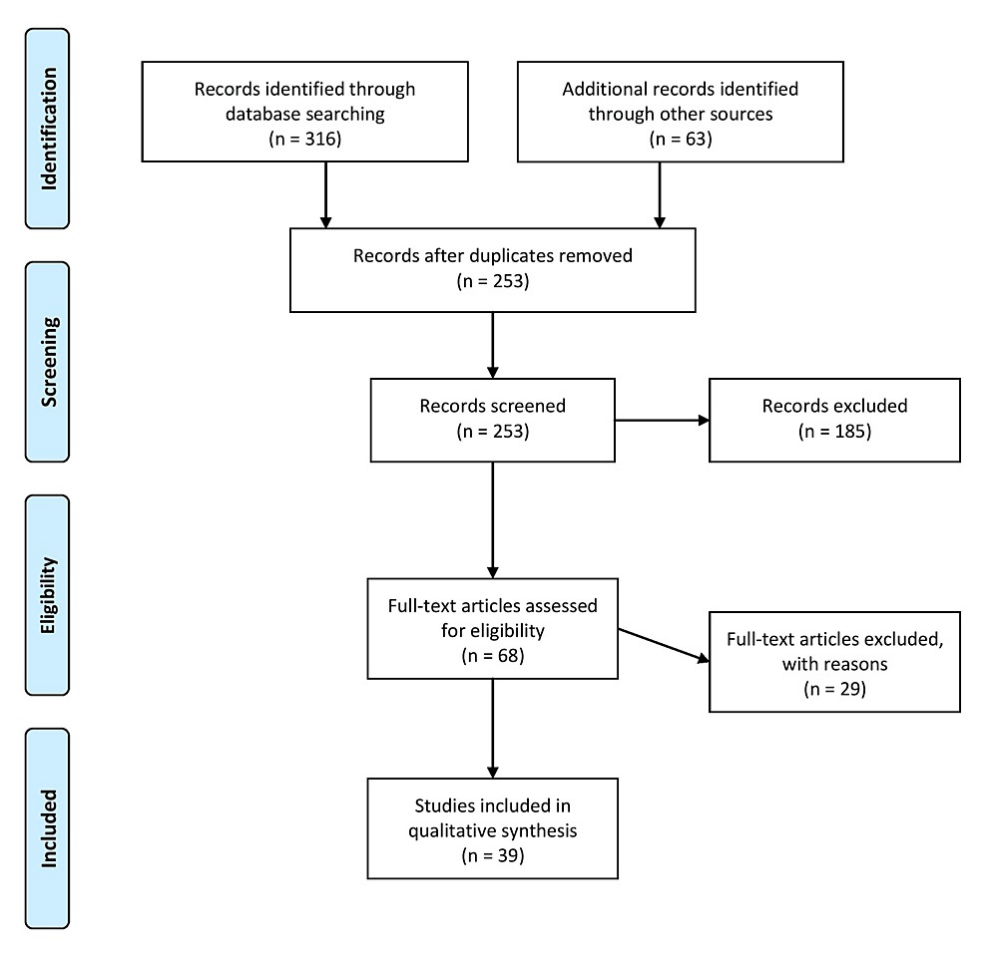
Selection process of articles used in this study. Adopted from the Preferred Reporting Items for Systematic Reviews and Meta-Analyses (PRISMA).

A comprehensive search was conducted in major medical databases, including PubMed, Scopus, and Web of Science, to identify relevant articles on using ChatGPT in radiology for medical imaging diagnosis. The search used keywords, such as “ChatGPT,” “artificial intelligence,” “radiology,” and “medical imaging diagnosis.” The search was limited to articles published in English from 2010 to 2023. To ensure the quality and relevance of the review, the review process followed a blind approach. This means that the identities of the authors and reviewers were concealed from one another to minimize bias. The blinding process helps maintain objectivity while evaluating the articles and prevents any potential influence from personal connections or reputations. The authors worked collaboratively throughout the review process. They collaborated on tasks, such as study selection, data extraction, quality assessment, and synthesis of findings. This collaborative approach allows for a comprehensive evaluation of the articles and helps ensure that the final review represents a consensus among the authors. Articles were included in the review if they discussed the use of ChatGPT in radiology and provided insights into its advantages and limitations for medical imaging diagnosis. The review encompassed different types of studies, including case studies, observational studies, and randomized controlled trials. Articles that did not discuss the use of ChatGPT in radiology, duplicates of previously identified articles, and non-peer-reviewed articles were excluded from the review. Figure 1 in the study describes the selection process used to identify the articles included in the review. The figure visually represents the steps to narrow the initial search results and select the most relevant articles for the review.

Advantages of ChatGPT in radiology

Improved Accuracy and Efficiency in Diagnosing Medical Images

ChatGPT's ability to analyze and interpret medical images using NLP algorithms is a major advantage in radiology. In traditional radiology, human radiologists must manually analyze medical images and interpret their findings, which can be time-consuming and error-prone. However, to leverage ChatGPT's accuracy, it requires specific prompts tailored to the medical images being analyzed [5,6]. By providing detailed prompts that include relevant information about images, such as the patient's medical history, symptoms, and image characteristics, ChatGPT can effectively process and interpret the images in real time, resulting in quick and accurate diagnoses. This innovative approach not only reduces the workload of human radiologists but also enhances the overall efficiency and reliability of the diagnostic process [5,6].

ChatGPT's algorithms are designed to recognize patterns in medical images and interpret them in a way that is similar to how human radiologists would do. The algorithm can analyze a large amount of data from medical images to identify patterns and anomalies and can even learn from previous cases to improve accuracy [7-9].

ChatGPT can help identify even the most subtle abnormalities in medical images that might be missed by human radiologists. Doing so can help improve patient outcomes by ensuring that patients receive accurate and timely diagnoses. In addition, ChatGPT can help reduce the need for additional testing and treatment, as the accuracy of its diagnoses can help prevent unnecessary testing and procedures [8,10,11].

Reduction in Interpretation Variability and Errors

Interpretation variability and errors are common issues in radiology that can occur due to differences in individual radiologists' interpretation of medical images. These differences can arise due to variations in training, experience, and personal biases, leading to inconsistencies in diagnosis that can impact patient outcomes [12].

One of the advantages of using ChatGPT in radiology is its ability to reduce interpretation variability and errors. This is because ChatGPT's algorithms are trained on large amounts of data, allowing it to learn patterns and interpret medical images with greater consistency and accuracy. ChatGPT is also not subject to the same human biases and cognitive limitations that can affect traditional radiologists [13].

ChatGPT can help reduce the variability and errors associated with traditional radiological diagnoses by providing more consistent and accurate diagnoses. This, in turn, can lead to better patient outcomes and improve the overall quality of care provided. In addition, ChatGPT can help radiologists focus on more complex cases, which may require a higher level of expertise and attention [14].

Improved Workflow Efficiency and Patient Outcomes

Improved workflow efficiency and patient outcomes can be achieved using ChatGPT in radiology for medical imaging diagnosis. ChatGPT can quickly analyze and interpret medical images in real time, reducing the time and resources required for diagnosis. This saves the radiologists time and helps reduce patient waiting times, leading to an overall improvement in patient experience [15,16].

Reduced waiting times allow patients to receive their diagnosis and treatment faster, leading to better patient outcomes. Early diagnosis of conditions, such as cancer, can significantly improve survival rates, and ChatGPT can help achieve this [17].

Moreover, with improved workflow efficiency, healthcare providers can serve more patients in a shorter amount of time, which can result in reduced healthcare costs. ChatGPT can also help reduce the need for repeat imaging studies, as it is less prone to interpretation errors, which can also save costs [2,15].

Limitations of ChatGPT in radiology

Data Quality and Quantity Limitations

ChatGPT's performance in radiology heavily depends on the quality and quantity of data it is trained on. The algorithm needs high-quality data to learn and develop the necessary patterns and features to diagnose medical images accurately. However, obtaining high-quality data can be challenging due to various factors, such as data privacy and security concerns, limited access to medical imaging data, and variations in data acquisition protocols [18].

Moreover, the data used to train ChatGPT must be diverse enough to represent the broad range of conditions and imaging techniques encountered in radiology. If the data are biased or limited in scope, they can affect the accuracy and reliability of ChatGPT's diagnoses. For example, if the training data predominantly represent one population or ethnicity, ChatGPT may not perform as well on images of patients from other populations. Similarly, if the data primarily represent images from certain imaging modalities, ChatGPT may struggle to accurately diagnose images from other modalities [19].

In addition, ChatGPT's performance may be limited in cases where it has not been trained on specific image types or conditions. For instance, ChatGPT may have difficulty detecting rare or novel conditions not included in the training data. Similarly, if the training data do not include images from specific subgroups, such as pediatric or elderly patients, ChatGPT may not perform as well on these images [7,8,20].

Ethical Considerations and Concerns Around AI's Role in Medical Decision-Making

Ethical considerations and concerns around the use of AI in medical decision-making are becoming increasingly important as AI technologies are being integrated into healthcare systems. AI algorithms, such as ChatGPT, may introduce new ethical challenges related to bias, accountability, and transparency [21].

For example, AI algorithms may be biased based on the data used to train them, leading to potential disparities in medical care. This is especially concerning in radiology, where inaccurate diagnoses could have serious consequences for patients. Furthermore, using AI in medical decision-making raises questions about accountability and transparency. It may be difficult to understand how the algorithm arrives at its decision, which makes it challenging for healthcare providers to explain decisions to patients [22,23].

Using factual information from various sources in ChatGPT may raise concerns regarding copyright issues. To address this, it is imperative to implement appropriate measures. These measures could include obtaining proper permissions and licenses for copyrighted materials, adhering to fair use policies, and ensuring that the use of copyrighted content falls within the boundaries of applicable copyright laws. By doing so, ChatGPT can mitigate any potential copyright infringement issues and operate in a legally compliant manner. Furthermore, when considering the integration of ChatGPT in radiological diagnoses, it is crucial to carefully evaluate the potential risks and benefits associated with its use. It is essential to establish robust ethical standards and regulatory frameworks to govern the deployment of ChatGPT in healthcare settings. These standards should encompass a range of considerations [24].

First, the training data utilized to develop the algorithm should be diverse and representative of the population it aims to serve. This ensures that the algorithm learns from various cases and demographics, minimizing biases and promoting equitable healthcare outcomes. Second, subjecting the algorithm to rigorous testing for fairness and accuracy is vital. Fairness testing ensures that the algorithm does not exhibit discriminatory behavior or biased outcomes across different demographic groups. Accuracy testing verifies the algorithm's performance against established standards and benchmarks, validating its reliability as a diagnostic tool. In addition, transparency plays a significant role in gaining trust and acceptance from healthcare providers. It is crucial to ensure that the algorithm's decision-making process is transparent and understandable. This can be achieved by developing explainable AI techniques, allowing healthcare professionals to comprehend how the algorithm arrives at its diagnoses and treatment recommendations [23-24].

Challenges and opportunities for future development

ChatGPT's potential for improving radiological diagnoses and patient outcomes is significant, but there are also challenges to consider. Some of these challenges include the following:

Limited Availability of High-Quality Training Data

In the context of ChatGPT's use in radiology, the accuracy and reliability of its diagnoses largely depend on the quality and quantity of the training data it receives. ChatGPT uses machine learning algorithms to learn patterns and make predictions based on the input data it receives during training. Developing new approaches to data generation, curation, and validation can help overcome this limitation and improve the accuracy and reliability of ChatGPT's diagnoses. However, obtaining high-quality training data for ChatGPT can be challenging in certain cases, particularly for rare or complex medical conditions. For example, rare diseases or unusual presentations of common diseases may not be represented in the available training data, which can result in inaccurate or unreliable diagnoses by ChatGPT [25].

In addition, even when high-quality training data are available, they may not always be accessible due to privacy concerns, data ownership, or limited availability of data-sharing resources. This can limit the ability of researchers and practitioners to develop and train ChatGPT models that can effectively diagnose a wide range of medical conditions [26,27].

As such, the limited availability of high-quality training data can be a significant limitation to the performance of ChatGPT in radiology, and efforts to improve data sharing and access, as well as the development of new approaches to data generation, curation, and validation, can help overcome this limitation and improve the accuracy and reliability of ChatGPT's diagnoses [26,27].

Need for Further Research and Development

It is important to address the limitations of ChatGPT in terms of higher-order skills, such as description, application, and classification. Further research and development are necessary to enhance these aspects of ChatGPT in the context of radiology for medical imaging diagnosis. While the current findings indicate potential, expanding the scope of images and conditions that ChatGPT can effectively diagnose is crucial. Although promising results have been observed in the analysis of CT scans and X-rays, it is necessary to explore and improve its ability to diagnose other types of medical images and conditions [15].

In addition, it is important to address potential biases in the training data that ChatGPT is trained on. Machine learning algorithms, including ChatGPT, are only as good as the data they are trained on. Therefore, it is critical to ensure that the data used to train ChatGPT are diverse, representative, and free from any biases that could potentially impact its performance and accuracy [5,28].

Finally, improving the explainability of ChatGPT's diagnoses is also a key area of focus for future research and development. While ChatGPT has shown great promise in improving the accuracy and efficiency of medical image diagnoses, its inner workings can be difficult to interpret and understand. Therefore, it is important to develop ways to explain how ChatGPT arrives at its diagnoses transparently and understandably so that clinicians and patients can have confidence in its results [4,29].

Ethical and Regulatory Considerations

As AI technologies, such as ChatGPT, are increasingly used in healthcare, it is important to consider their ethical and regulatory implications. One key consideration is ensuring that the use of ChatGPT is fair and transparent in its decision-making processes. It is essential to ensure that the algorithms used by ChatGPT do not perpetuate biases that can harm certain groups, such as those based on race, gender, or socioeconomic status. To achieve this, ChatGPT should be trained on diverse datasets, and its performance should be continually evaluated to identify and address potential biases [30].

In addition to addressing patient privacy concerns, it is crucial to address the ethical implications of making healthcare more accessible to inaccessible and underserviced areas. By leveraging ChatGPT in radiology, we can extend the reach of medical expertise to areas that lack sufficient healthcare resources, providing timely and accurate diagnostic support. This has the potential to improve patient outcomes and reduce health disparities significantly. To ensure ethical implementation, it is paramount to establish guidelines and protocols that prioritize equitable access to healthcare. Initiatives should be in place to identify and prioritize underserved regions and populations, ensuring that they receive adequate attention and support. In addition, efforts should be made to involve local healthcare professionals in the decision-making process, allowing their insights and expertise to shape the deployment of ChatGPT in these areas [31].

While improving accessibility, it is crucial to remain committed to safeguarding patient privacy and confidentiality. Strict measures must be in place to protect sensitive medical data and ensure it is used solely for its intended purpose. Robust security protocols should be implemented to prevent unauthorized access or breaches, and regular audits should be conducted to identify and rectify potential vulnerabilities. By addressing both the ethical implications and patient privacy concerns, we can harness the power of ChatGPT in radiology to bridge the healthcare gap and provide quality care to previously underserved areas. This balanced approach will contribute to a more equitable healthcare system while maintaining safeguards to protect patient information [30-31].

In addition, the use of AI in medical decision-making raises important ethical questions about the role of technology in patient care. It is crucial to ensure that ChatGPT is in line with accepted medical practices and is used ethically and responsibly to support, rather than replace, human decision-making [31].

To address the ethical and regulatory concerns surrounding the use of ChatGPT in radiology, it is imperative to establish clear and well-defined guidelines and standards. Regulatory bodies and professional organizations should actively contribute to developing these guidelines and take measures to ensure their implementation by medical professionals and healthcare organizations. Ongoing monitoring, evaluations, and open dialogues among healthcare professionals, patients, and other relevant stakeholders are essential to ensure the responsible use of ChatGPT in radiology, maintain its ethical nature, and optimize patient care [32].

Despite these challenges, there are also significant opportunities for the future development of ChatGPT in radiology, including the following:

Improved Accuracy and Efficiency in Radiological Diagnoses

ChatGPT's ability to analyze and interpret medical images in real time can significantly improve the accuracy and efficiency of radiological diagnoses. Traditional methods of interpreting medical images, such as X-rays, CT scans, and MRI scans, can be time-consuming and may require multiple specialists to interpret the images. This can lead to a delay in diagnosis, increased interpretation variability, and higher healthcare costs. ChatGPT's ability to quickly analyze and interpret medical images can help reduce the time and resources required for interpretation, leading to faster and more accurate diagnoses [33].

This can lead to improved patient outcomes faster and more accurate diagnoses can lead to improved patient outcomes, as early diagnosis and treatment can help prevent disease progression and improve health outcomes. ChatGPT's ability to quickly analyze medical images can also help identify smaller or more subtle abnormalities that may be missed by human interpretation. This can lead to earlier detection of diseases and conditions, improving patient outcomes and potentially saving lives [34].

ChatGPT in radiology can reduce healthcare costs by reducing the time and resources required for interpretation. This can also help reduce the number of unnecessary follow-up appointments and tests, as more accurate diagnoses can help avoid misdiagnosis or missed diagnoses. In addition, faster and more accurate diagnoses can help reduce the need for invasive procedures, further reducing healthcare costs [5,14].

Integration With Other Technologies

ChatGPT has the potential to be integrated with other healthcare technologies, such as electronic health records (EHR) and telemedicine platforms. EHR systems are used to store patient health information, including medical history, test results, and medication lists, and are commonly used in healthcare settings. By integrating ChatGPT with EHR systems, medical imaging results can be directly incorporated into a patient's medical record, providing healthcare providers with a more comprehensive understanding of their patient's health [15,18].

Telemedicine platforms can also be integrated with ChatGPT, allowing for remote consultations and data exchange. This can be particularly useful for patients who live in rural or remote areas and have limited access to specialized medical imaging services. Telemedicine platforms allow patients to receive timely and accurate diagnoses without traveling long distances to see a specialist in person. This can improve patient access to care and reduce wait times for medical imaging services [35].

Personalized Medicine

Personalized medicine is a field of medicine that aims to provide individualized treatments tailored to the unique characteristics of each patient. ChatGPT's ability to analyze vast amounts of patient data can be instrumental in enabling personalized medicine by identifying patterns and trends in patient data that can inform treatment decisions [14].

With the help of ChatGPT, medical professionals can analyze complex patient data, including medical imaging results, genetic data, and clinical data, to identify potential disease risk factors, diagnose diseases, and develop personalized treatment plans. ChatGPT can enable more precise and accurate diagnoses, reduce diagnostic errors, and improve patient outcomes [36].

Moreover, ChatGPT can help medical professionals identify potential adverse drug reactions and recommend alternative treatments that are more effective for the individual patient based on their unique characteristics. This can reduce the risk of adverse drug reactions and improve patient safety [1,14].

The potential for ChatGPT to enable personalized medicine is particularly significant in oncology, where treatment decisions are complex and often require a multidisciplinary approach. By analyzing a patient's medical data, ChatGPT can help oncologists identify the most effective treatments and tailor them to their unique characteristics [18].

Need for Further Research and Development to Improve ChatGPT’s Performance and Usability

ChatGPT is a relatively new technology, and although it has shown promise in radiology, there is still much to be done to improve its performance and usability. One main area that requires further research and development is expanding the scope of the images and conditions that ChatGPT can diagnose. Currently, the system may be limited to certain types of medical images or conditions, and expanding its capabilities will require more extensive training data and ongoing development efforts [37,38].

Another concern is potential biases in the training data used to train ChatGPT. If the training data are not diverse enough or if there are underlying biases in the data, diagnoses could be inaccurate or incomplete. Therefore, further research and development efforts should ensure that the training data are representative and diverse [39].

Finally, improving the explainability of ChatGPT's diagnoses is crucial. As an AI system, ChatGPT cannot explain its diagnoses in the same way that a human radiologist can. This can be a significant challenge for physicians and patients who need to understand the basis for the diagnosis. Therefore, further research and development efforts should aim to improve the explainability of ChatGPT's diagnoses so that they can be more easily understood and communicated [18,28].

## Conclusions

ChatGPT has shown great promise in improving the accuracy and efficiency of radiological diagnoses. Its ability to analyze and interpret medical images in real time can help reduce interpretation variability and errors, improve workflow efficiency, and ultimately enhance patient outcomes. In addition to these benefits, ChatGPT has the potential for various other applications, such as medical education and differential diagnosis, which should be further elaborated upon. By leveraging ChatGPT's capabilities in medical education, healthcare professionals can use the system as a valuable tool for training and enhancing their knowledge in radiology. It can provide a platform for interactive learning, allowing users to ask questions, receive detailed explanations, and gain a deeper understanding of various imaging findings and pathologies. Moreover, incorporating ChatGPT into medical education programs can contribute to standardizing knowledge and disseminating up-to-date information across different institutions. Furthermore, ChatGPT can be utilized in differential diagnosis, aiding radiologists in the process of distinguishing between similar medical conditions based on imaging findings. By leveraging its ability to analyze a wide range of imaging data, ChatGPT can assist in generating a list of potential diagnoses, considering different factors and presenting them to the radiologist for further evaluation. This can help improve diagnostic accuracy and reduce the likelihood of missed or delayed diagnoses, leading to better patient care and outcomes. While ChatGPT offers promising advantages, it is essential to acknowledge certain limitations. High-quality training data are crucial for the system to perform effectively and reliably. Efforts should be made to ensure that the training data accurately represent the diverse range of imaging findings encountered in clinical practice. In addition, ethical considerations must be carefully addressed, including privacy concerns, patient consent, and transparency in AI decision-making processes.
